# Programmed Cell Death in Developing *Brachypodium distachyon* Grain

**DOI:** 10.3390/ijms22169086

**Published:** 2021-08-23

**Authors:** Safia Saada, Charles Ugochukwu Solomon, Sinéad Drea

**Affiliations:** 1 Department of Genetics and Genome Biology, University of Leicester, University Road, Leicester LE1 7RH, UK; ss1330@leicester.ac.uk (S.S.); sd201@le.ac.uk (S.D.); 2 Department of Plant Science and Biotechnology, Abia State University, Uturu PMB 2000, Nigeria

**Keywords:** *Brachypodium* *distachyon*, cereals, grains, programmed cell death, proteases

## Abstract

The normal developmental sequence in a grass grain entails the death of several maternal and filial tissues in a genetically regulated process termed programmed cell death (PCD). The progression and molecular aspects of PCD in developing grains have been reported for domesticated species such as barley, rice, maize and wheat. Here, we report a detailed investigation of PCD in the developing grain of the wild model species *Brachypodium distachyon*. We detected PCD in developing *Brachypodium* grains using molecular and histological approaches. We also identified in *Brachypodium* the orthologs of protease genes known to contribute to grain PCD and surveyed their expression. We found that, similar to cereals, PCD in the *Brachypodium* nucellus occurs in a centrifugal pattern following anthesis. However, compared to cereals, the rate of post-mortem clearance in the *Brachypodium* nucellus is slower. However, compared to wheat and barley, mesocarp PCD in *Brachypodium* proceeds more rapidly in lateral cells. Remarkably, *Brachypodium* mesocarp PCD is not coordinated with endosperm development. Phylogenetic analysis suggests that barley and wheat possess more vacuolar processing enzymes that drive nucellar PCD compared to *Brachypodium* and rice. Our expression analysis highlighted putative grain-specific PCD proteases in *Brachypodium*. Combined with existing knowledge on grain PCD, our study suggests that the rate of nucellar PCD moderates grain size and that the pattern of mesocarp PCD influences grain shape.

## 1. Introduction

Ordered and finely regulated elimination of cells in living organisms is called programmed cell death (PCD).

PCD events that occur during grain development have been reported in cereals. Generally, the nucellus rapidly undergoes PCD in the first five days following fertilization, generating space and leaving behind growth materials for the incipient endosperm [1,2,3]. In maize, nucellus PCD proceeds acropetally while in barley, rice, and wheat, nucellus PCD is centrifugal [4,5,6]. All that remains of the nucellar tissues at about 6 days post anthesis (DPA) are the nucellar epidermis, which is bordered on the outside by the inner integument; nucellar lysate, which is debris of lysed nucellus cells sandwiched between the nucellar epidermis and the expanding endosperm; and the nucellar projection, which is located at the chalazal region between the endosperm and pigment strand [2,3,7,8,9,10]. In barley and wheat, the nucellar epidermis persists for a longer period during grain development, retaining cytological integrity and metabolic activity until midway through grain development [3,11]. In rice the nucellar epidermis serves as a transport route for nutrients into the endosperm and eventually collapses by 21 DPA, terminating grain filling [9,12,13]. PCD in the nucellar projection region proceeds from cells proximal to the endosperm towards cells at the chalazal region [10,14,15]. Nucellar projection PCD has been detected at 3, 8 and 13 DPA in rice, barley and wheat, respectively [5,6,15,16].

At anthesis, the cereal pericarp, from outside to inside, is composed of a layer of cutinous epicarp, several layers of parenchymal mesocarp and two to three layers of chlorenchymal endocarp. PCD occurs in barley and rice mesocarp cells closer to the integuments at 6 DPA and proceeds in an outward pattern. By 15 DPA, the mesocarp and endocarp have undergone PCD, leaving behind two to three layers of empty cells crushed between the outer cuticle and the integument [1,8,13]. In contrast to nucellar and pericarp cells, which disintegrate after PCD, endosperm cells remain intact after undergoing PCD [1]. Rice endosperm PCD occurs in a centrifugal pattern and was detected by 8 DPA [13,17]. PCD was detected in maize and wheat endosperm by 16 DPA. Maize endosperm PCD proceeds in a basipetal pattern while PCD proceeds randomly in wheat endosperm [18].

Multiple families of proteases have been linked with PCD in developing grains. The expression profile of nucellin, an aspartic protease that belongs to Clan AA Family A1 (http://merops.sanger.ac.uk, accessed on 10 April 2019), highly correlates with the degeneration of the nucellus in barley and rice, suggesting that it has a role in nucellus PCD [19,20]. Another known aspartic protease, *Oryzasin* (*Os05g0567100*), was found to be expressed during rice seed ripening and germination [21]. Notably, cysteine proteases belonging to the legumain family (C13), also known as vacuolar processing enzymes (VPEs), have been shown to be expressed in a temporal- and tissue-specific manner that coincides with caspase-like activities in developing barley grains [5,22,23,24,25,26]. The mRNA of barley nucellain orthologs was shown to localize in situ on nucellar cells undergoing PCD in wheat and *Brachypodium distachyon* [27,28]. Rice nucellain, *OsVPE1*, was significantly down-regulated in *OsMADS29*-knockdown transgenic seeds, which may indicate direct regulation of *OsVPE1* by *OsMADS29* in rice [29]. Recent functional characterization of *HvVPE4* by RNA interference revealed that pericarp PCD is inhibited by downregulation of *HvVPE4*, leading to reduced size and storage capacity in the embryo and endosperm [30]. The expression of a cathepsin B-like protease gene belonging to the papain family (C1) overlaps with PCD in the nucellus, nucellar epidermis and nucellar projection of developing wheat grains [11]. A rice Cys protease gene, *Os02g48450*, also belonging to the papain (C1) family, is severely downregulated in the nucellar projection of A-*OsMADS29* (*OsMADS29*-knockdown) lines. Promoter analysis of *Os02g48450* upstream sequence revealed clusters of CArG-box motifs that were shown experimentally to be bound by *OsMADS29* [6]. The rice MADS box family transcription factor *OsMADS29*, an ortholog of Arabidopsis thaliana TRANSPARENT TESTA 16 (*TT16*), regulates PCD in the pericarp, ovular vascular trace, integuments, nucellar epidermis and nucellar projection during rice grain development. Suppression of *OsMADS29* expression either by antisense constructs or RNA interference resulted in partially filled grains that were small and shrunken in shape. The knockdown mutant grains also had smaller endosperm cells, reduced starch synthesis and abnormally shaped starch granules [6,29,31]. 

*Brachypodium distachyon* (subsequently *Brachypodium*) was first proposed as a model system for grasses [32]. It is phylogenetically related to wheat, barley and rye. Mature *Brachypodium* grains have less starch in thick-walled endosperm cells compared to cereal grains. This is unusual because *Brachypodium* expresses the full set of genes required for starch synthesis in its endosperm [33]. Other aspects of grain development in *Brachypodium* have been reported [28,33,34,35,36,37,38,39]. However, details of PCD events in developing *Brachypodium* grains, to the best of our knowledge, have not yet been reported.

Hands et al. [38] revealed that *Brachypodium* grains have an enlarged persistent nucellar epidermis at maturity. This contrasts with cereal grains, where degeneration of the nucellar epidermis and other maternal tissues leaves room and provision for the expanding endosperm. We hypothesized that the pattern and progression of PCD in *Brachypodium* grains may be different from cereals. Here, we tested this hypothesis by conducting a systematic histochemical and molecular study of PCD in developing *Brachypodium* grains. Our results suggest that the timing and pattern of PCD may underlie differences in grain size and shape between wild and domesticated species.

## 2. Results

### 2.1. TUNEL and Vital Staining Reveal Pattern and Progression of PCD in Developing Brachypodium Grain

**Figure 1:**
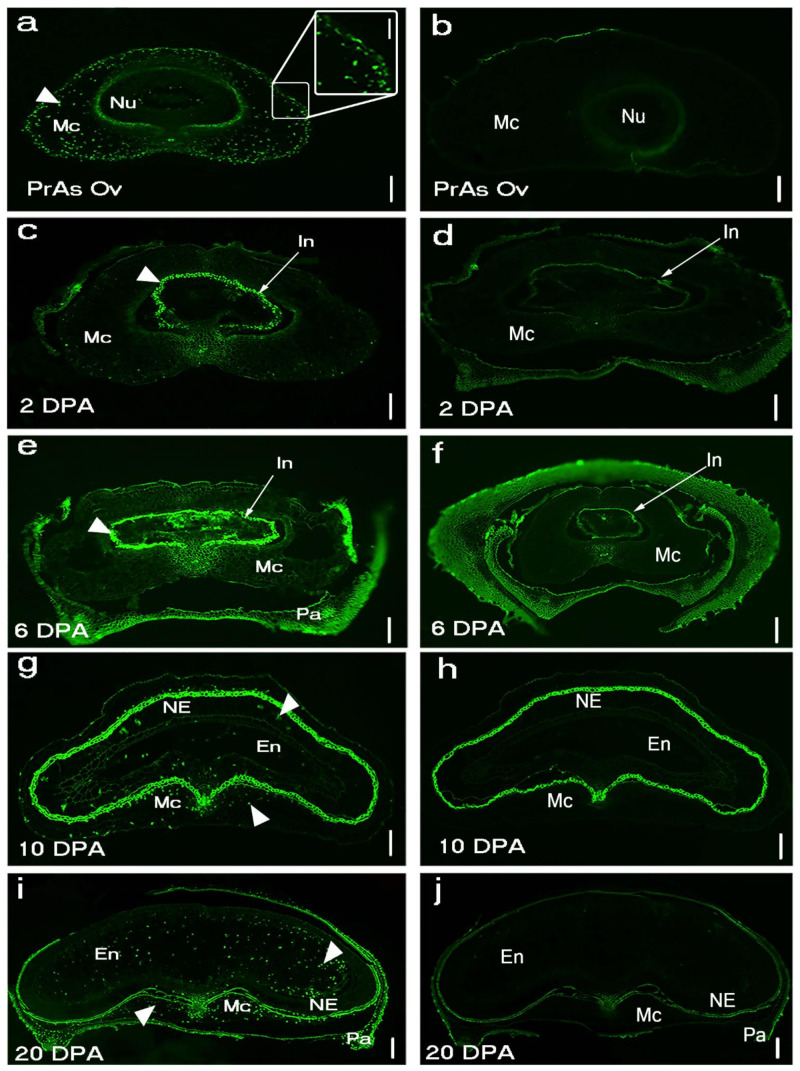
DNA fragmentation detected in *Brachypodium* grain section using TUNEL assay in five developmental stages: (**a**) Pr As Ov: pre-anthesis ovary; (**c**) young grain at 2 days post-anthesis (2 DPA); (**e**) mid-length grain at 6 DPA; (**g**) full-length grain at 10 DPA; (**i**) mature grain at 20 DPA; (**b**,**d**,**f**–**h**) negative control. Arrowheads indicate DNA fragmentation signals. The bright signal around the nucellar epidermis in (**g**) is autofluorescence from the integuments. NE: nucellar epidermis; Nu: nucellus parenchyma cells; Mc: mesocarp’ Pa: palea, In: integument; En: endosperm. Scale bar (**a**–**j**) 100 µm, (**a**) insert 40 µm.

**Figure 2:**
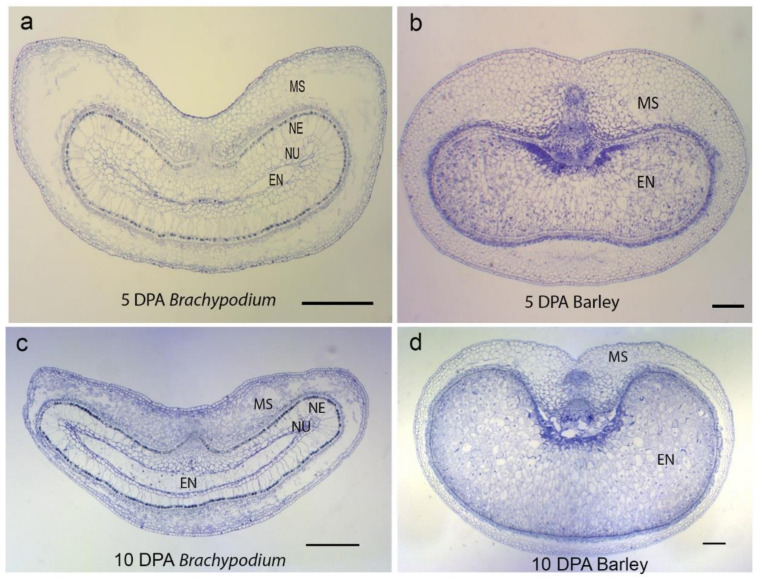
Comparison of grain development in *Brachypodium* and barley observed with light microscopy stained with toluidine blue. At 5 DPA, (**a**) *Brachypodium* endosperm is barely differentiated and is surrounded by several layers of nucellar cells. Additionally, the mesocarps have largely undergone PCD, especially on the lateral sides. (**b**) Barley endosperm is fully differentiated and set for filling. Nucellar cells appear absent and the mesocarp cells remain intact. At 10 DPA, (**c**) *Brachypodium* nucellar epidermis persists and a few layers of nucellus surround the endosperm. Nucellar epidermis and nucellus appear absent in 10 DPA barley (**d**), and the endosperm occupies a large area of the grain. Note that the barley mesocarp has underdone PCD in a centrifugal pattern. Scale bars: 0.2 mm. EN, endosperm; MS, mesocarp; NE, nucellar epidermis; NU, nucellus.

**Figure A1:**
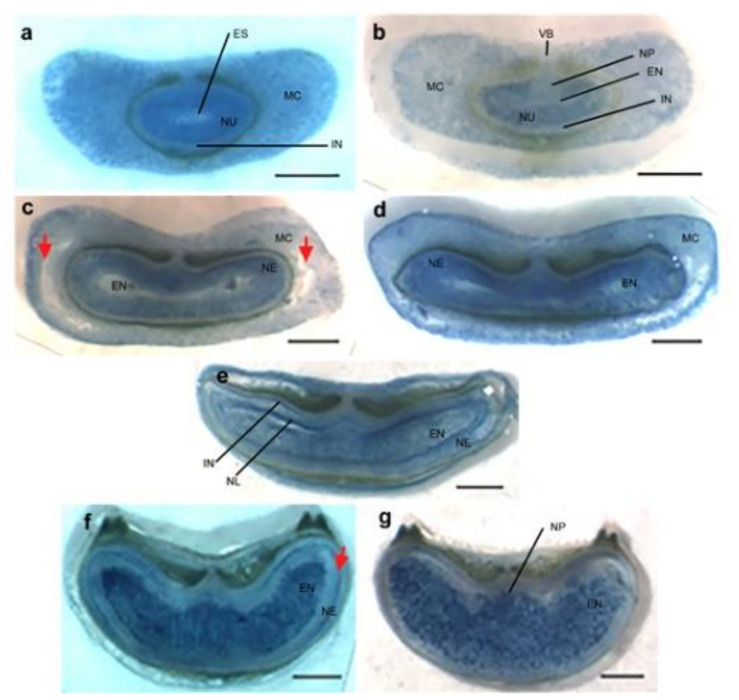
Vital staining of developing *Brachypodium* caryopsis. Freehand transverse sections of developing *Brachypodium* grains stained with 0.05% Evans Blue. (**a**) The nucellus and mesocarp cells stained intensely in pre-anthesis ovary. **(b**) Two days post anthesis (DPA), the nucellar projection, endosperm and integument are viable and not stained. (**c**) 6 DPA, red arrows indicate degenerating lateral mesocarp cells. (**d**) 10 DPA, the nucellar epidermis is deeply stained indicating PCD. (**e**) 15 DPA, the integuments and nucellar lysate are stained. Endosperm cells are randomly stained. (**f**) 20 DPA, staining of the transport channel through the nucellar epidermis is indicated by a red arrow. (**g**) 25 DPA, endosperm and nucellar epidermis are deeply stained. Scale bars: 0.2 mm. EN, endosperm; ES, embryo sac; IN, integument; MC, mesocarp; NE, nucellar epidermis; Nu, nucellar; NL, nucellar lysate; NP, nucellar projection; VB, vascular bundle.

TUNEL-positive signals and Evans blue staining of sections made from unfertilized *Brachypodium* ovary (pre-anthesis ovary) (Figure 1a,b and Figure A1) suggested that PCD of nucellar and mesocarp cells precedes fertilization. Nucellus PCD progressed in a centrifugal pattern post-fertilization. Although TUNEL-positive signals and Evans blue staining suggested rapid nucellus PCD, post-mortem clearance of dead nucellus cells appeared to progress slowly. Whereas we observed complete clearance of nucellus cells at the 5 DPA in barley, four to six layers of nucellus cells were still present in 5 DPA *Brachypodium* grain (Figure 2). The nucellar epidermis of *Brachypodium* grains serves as an assimilate transport route during grain filling and is greatly enlarged by 5 DPA (Figure 2a) [40]. TUNEL and Evans blue staining indicated nucellar epidermis PCD at 10 DPA (Figure 1g,h and Figure A1d). Integument PCD was detected as early as 2 DPA and appeared to continue up to 6 DPA (Figure 1c–f). 

**Figure A2:**
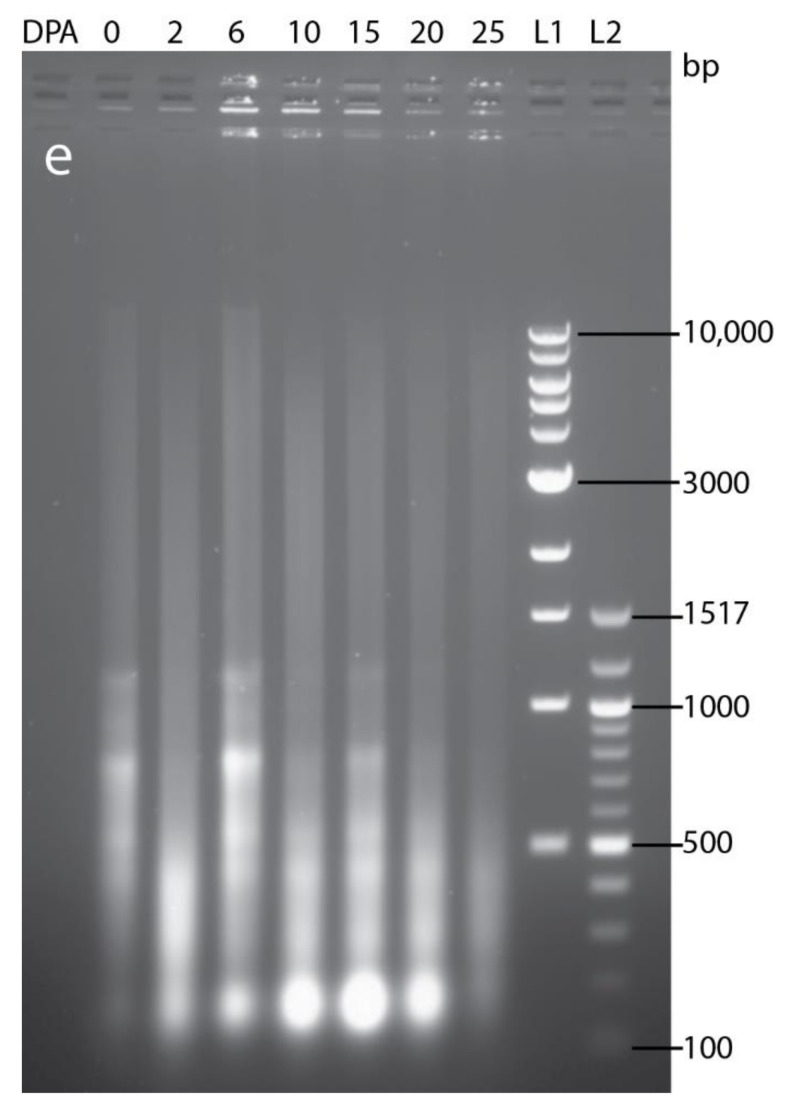
DNA fragmentation of developing *Brachypodium* grains. DNA was extracted from pre-anthesis ovary (0). Grains were sampled on various days post anthesis (DPA), as indicated above the lanes, and separated on 1% agarose gel. The sizes of 1 kb (L1) and 100 bp (L2) ladders are shown.

PCD of cells that make up the post-phloem transport pathway (pigment strand and nucellar projection) was not detected by Evans blue staining before 25 DPA (Figure A1b–g). This observation agrees with their known role in assimilate transport to the endosperm during grain filling. DNA fragmentation was uniformly detected in the mesocarp of *Brachypodium* pre-anthesis ovary (Figure 1a). However, mesocarp cell disintegration proceeded most rapidly in lateral cells (Figure 2a and Figure A1c). Endosperm PCD, indicated by positive Evans blue stain, was detected by 15 DPA in a random pattern and increased in intensity thereafter (Figure 1i,j and Figure A1d). At 25 DPA, the entire endosperm was clearly stained except the aleurone layers. The progression of DNA fragmentation from pre-anthesis ovary to grain maturity is also shown by DNA laddering using gel electrophoresis (Figure A2). From to pre-anthesis 20 DPA there was an increase in the amount of DNA fragments with a length of less than 200 nt. This suggests that DNA cleavage is happening throughout grain development, though in different tissues. Less DNA fragments were detected at 25 DPA, by which time the grain is mature and the tissues that remain viable (embryo and aleurone) are not undergoing PCD.

### 2.2. Potential Involvement of MADS29 in Brachypodium PCD

**Figure 3:**
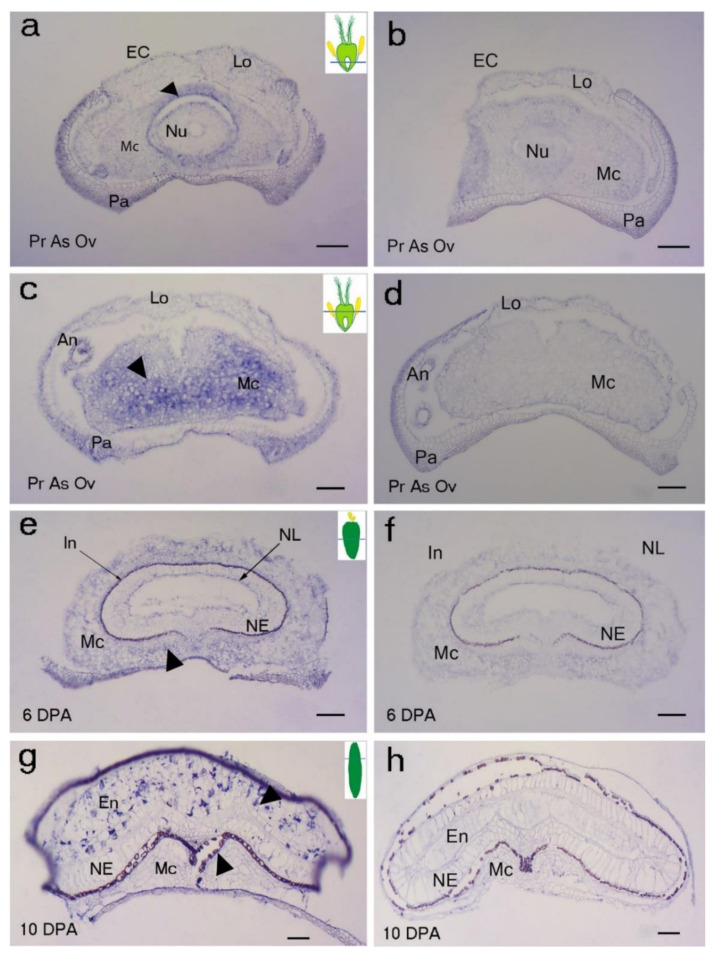
Localization of *BdMADS29* transcript in developing grain sections of *Brachypodium distachyon*. (**a**–**d**) Pr As Ov: pre-anthesis ovary; (**e**) mid-length grain at 6 DPA; (**g**) mature grain at 20 DPA; (**a**–**c**,**e**–**g**) antisense; (**b**–**d**,**f**–**h**) sense. Arrowheads indicate hybridization signals. An: anther; Fi: filament; NE: nucellar epidermis; Nu: nucellus parenchyma cells; Lo: lodicule; Mc: mesocarp; Pa: palea; Ec: endocarp; In: integument; En: endosperm; NL: nucellar lysate. Scale bar 100 µm.

Due to the importance of *OsMADS29* during rice grain PCD [6], we selected its ortholog in *Brachypodium* and analysed its localisation in developing *Brachypodium* grains using mRNA in situ hybridisation (Figure 3). *BdMADS29* expression was detected in the endocarp and mesocarp cells above the ovule at pre-anthesis ovary (Figure 3a–f). We found that *BdMADS29* generally localizes in cells undergoing PCD in developing *Brachypodium* grain and other tissues. Importantly, *BdMADS29* also localized in *Brachypodium* endosperm cells undergoing PCD (Figure 3g,h). In contrast, *OsMADS29* was not associated with rice endosperm PCD [6]. 

### 2.3. Brachypodium Lacks Expansion of VPEs Found in Triticeae

**Figure 4:**
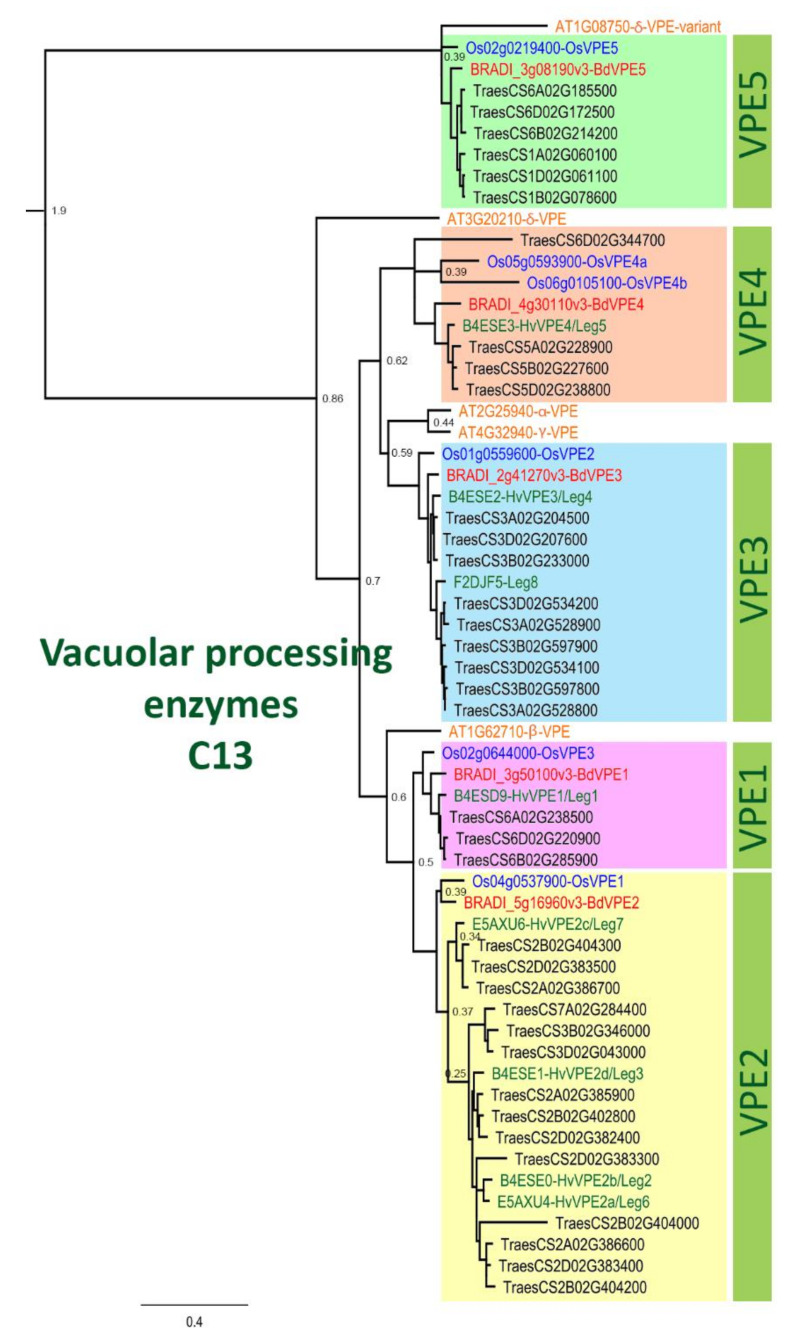
Phylogenetic tree of vacuolar processing enzymes of the C13 family. This tree illustrates the five groups VPE1-5 and includes VPE proteins from Arabidopsis (orange font), rice (blue font), *Brachypodium* (red font), barley (green font) and wheat (black font). JTT+I was used as protein model and the sequences were aligned with ClustalW, then inferred using the maximum likelihood method with a bootstrap of 1000 replicates. The tree was generated using MEGA10. The node numbers indicate the bootstrap value.

**Figure 5:**
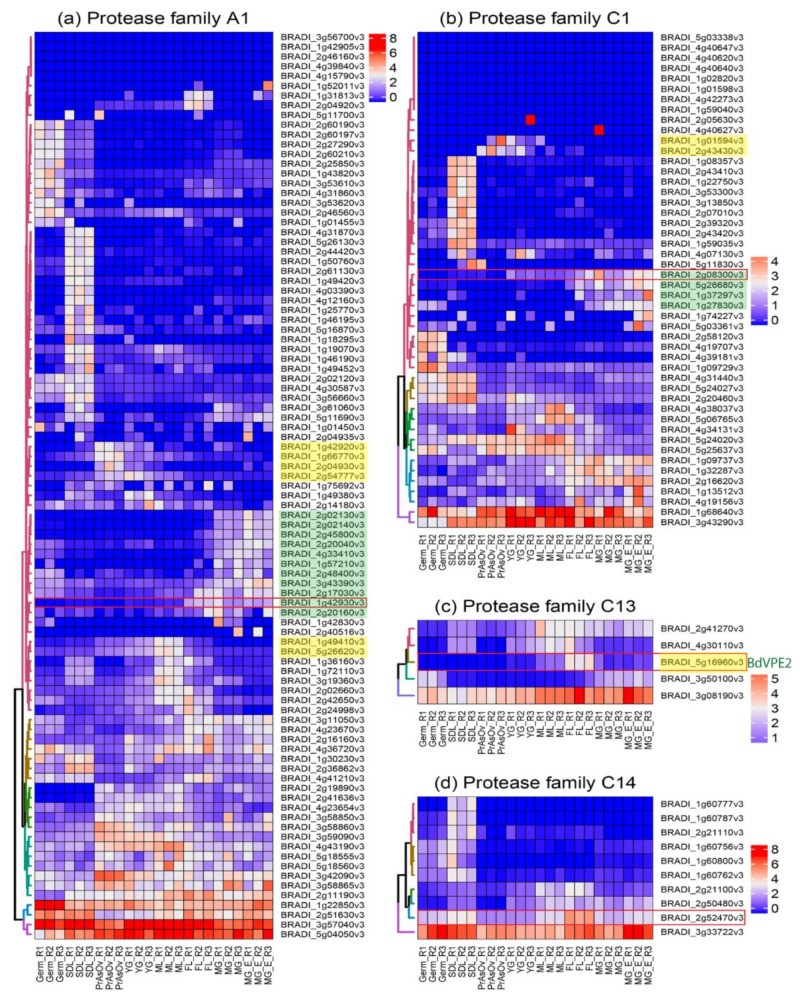
Hierarchically clustered expression profile of protease genes from (**a**) A1, (**b**) C1, (**c**) C13 and (**d**) C14 families in vegetative and grain tissues of *Brachypodium*. Heatmap was plotted with DESeq2 normalized RNA-Seq reads centered with unit variance scaling. Genes in clusters highlighted are active in nucellar tissue and the mesocarp (yellow), and the endosperm (green). Red rectangles indicate the genes used in qPCR for validation. PrAnOv, pre-anthesis ovaries; YG, young grain (1–3 DPA); ML, mid-length grain (3–8 DPA); FL, full-length grain (8–15 DPA); MG, mature grain (15–20 DPA); MGE, mature grain without embryo; Germ, germinating grain; SDL, seedling 3–4 days after germination.

**Figure A3:**
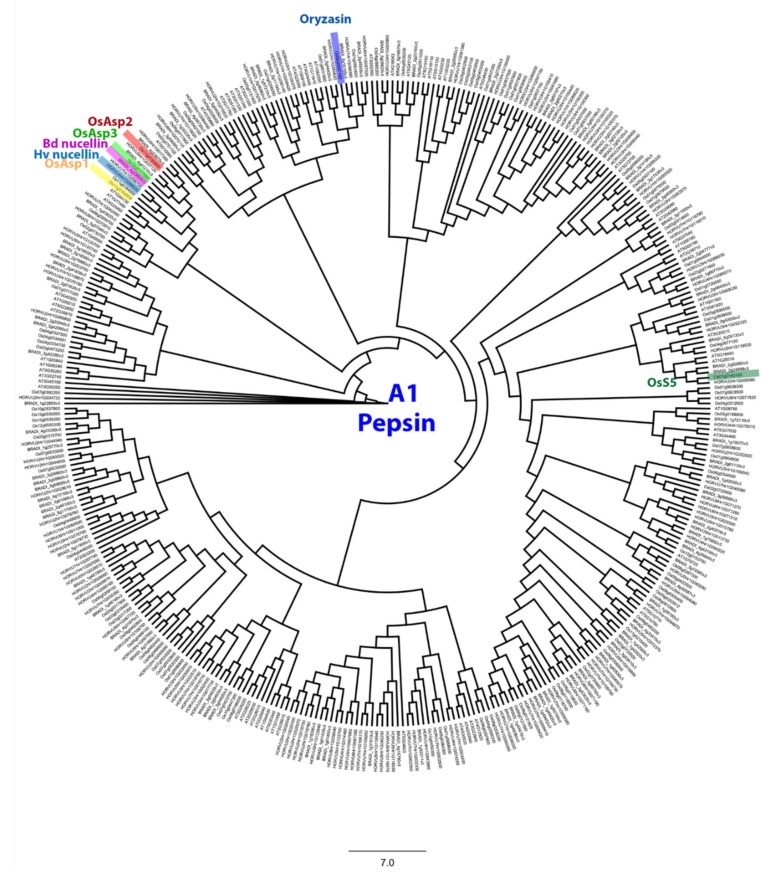
Phylogenetic tree of the pepsin-like A1 family. This tree includes aspartic protease from *Arabidopsis thaliana*, *Oryza sativa*, *Brachypodium distachyon* and *Hordeum vulgare*. Identified genes in Hv, Bd and Os were highlighted. JTT+G was used as protein model and the sequences were aligned with MAFFT v7.308 [57], then inferred using the maximum likelihood method with a bootstrap of 1000 replicates. The tree was generated using Geneious R10.

**Figure A4:**
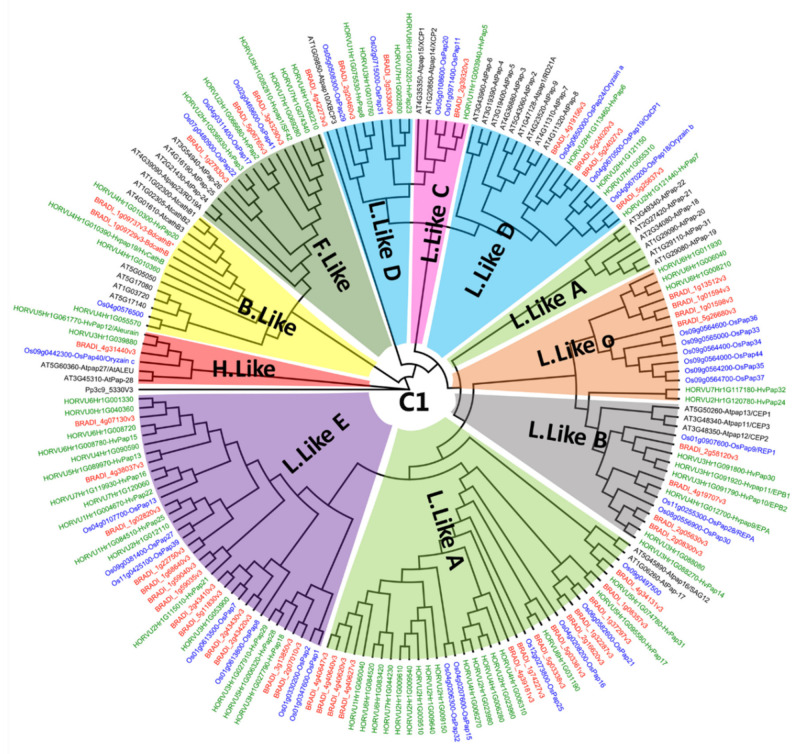
Phylogenetic tree of the papain C1 family. This tree includes protease from *Arabidopsis thaliana*, *Oryza sativa*, *Brachypodium distachyon* and *Hordeum vulgare*. Sequences were grouped in nine sub-clades (B.Like, C.Like, F.Like, H.Like and 5 type of L.Like). JTT+G was used as a protein model and the sequences were aligned with MAFFT v7.308 [57], then inferred using the maximum likelihood method with a bootstrap of 1000 replicates. The tree was generated using Geneious R10.

**Figure A5:**
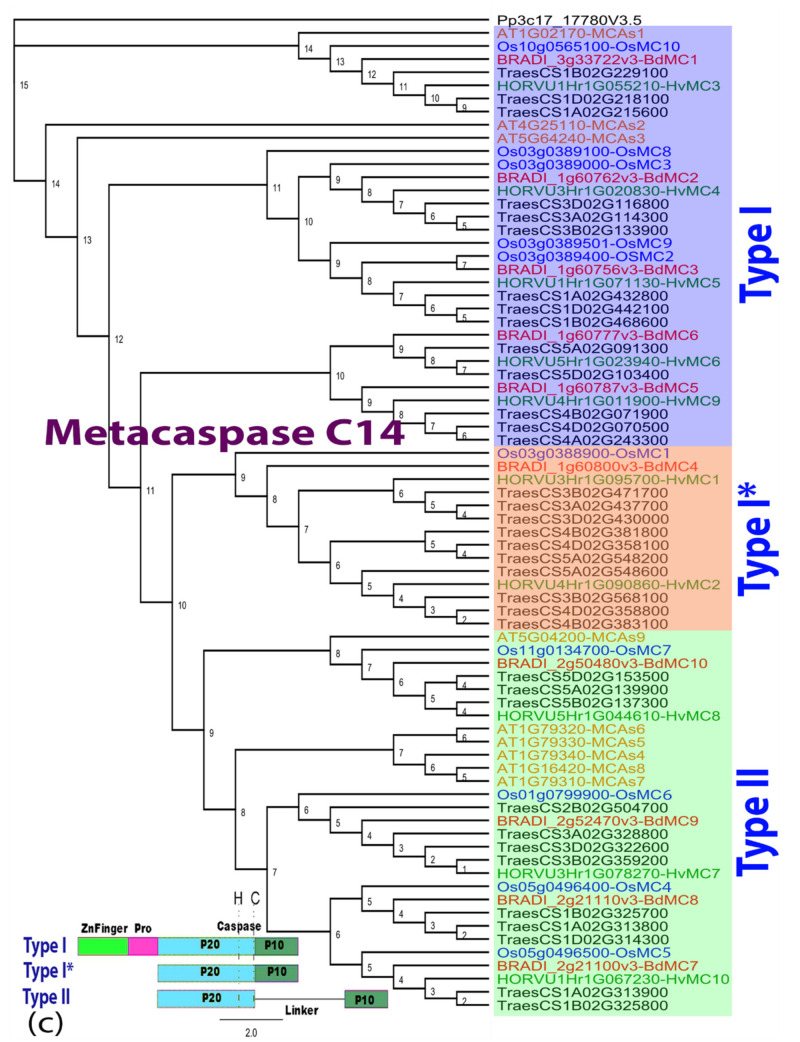
Phylogenetic tree of matacaspase C14 family. This tree includes protease from *Arabidopsis thaliana*, *Oryza sativa*, *Brachypodium distachyon*, *Hordeum vulgare* and *Triticum aestivum*. Sequences were grouped in three types based on their sequence statures. JTT+G was used as a protein model and the sequences were aligned with MAFFT v7.30 [57], then inferred using the maximum likelihood method with a bootstrap of 1000 replicates. The tree was generated using Geneious R10. Pro: proline domain.

We performed phylogenetic analysis comparing PCD proteases in *Brachypodium* and other species. Our sequence search identified 93, 48, 5, and 10 genes belonging to *Brachypodium* A1, C1, C13 and C14 protease family genes, respectively (Table A1). Phylogenetic analyses incorporating protein sequences of these gene families from *Arabidopsis*, barley, rice and wheat enabled the identification and naming of putative orthologs in *Brachypodium*. Phylogenetic trees for A1, C1 and C14 families are presented in Figure A3, Figure A4 and Figure A5, respectively. The phylogenetic tree for C13 (Figure 4) shows that monocot VPE protein sequences included in the analysis cluster into five clades with one *Brachypodium* VPE protein in each clade. Interestingly, we observed an expansion of *Triticeae* (represented by barley and wheat) sequences in VPE2, VPE3 and VPE5 clusters that is absent in *Brachypodium* and rice. Barley and wheat have 4 and 14 sequences in the VPE2 clade, respectively (Figure 4). Three of the barley VPEs in that clade, *HvVPE2a*, *HvVPE2b* and *HvVPE2d*, are most highly expressed at four days after flowering, which coincides with nucellus PCD in barley endosperm fractions [5]. *BdVPE2* on the other hand clusters more closely to *HvVPE2c* whose expression was hardly detected in developing barley grains. We confirmed the expansion of VPE2 cluster by examining the Ensembl Plants gene tree that contains *BdVPE2* (data not shown), which showed that species in the *Triticeae* tribe have more sequences in this clade than species in *Brachypodeae*, *Oryzinae*, and *Panicoideae*. In addition, *BdVPE2* (*BRADI5g16960*) expression in our transcriptome data was highest in mid-length and full-length grains (Figure 5), a period that coincides with PCD in the mesocarp and endosperm and not the nucellus. This suggests functional differences between barley *HvVPE2* genes and *BdVPE2*.

### 2.4. Expression Analysis Identifies Putative Grain Specific Proteases 

**Figure 6:**
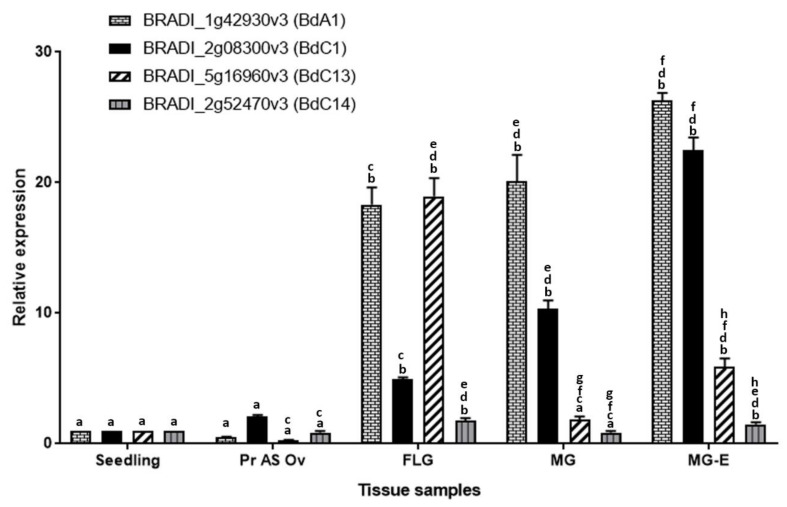
Normalized relative RT-qPCR expression of selected peptidase candidates, in seedling, pre-anthesis ovary (Pr As Ov). Bars with same alphabets (**a–h**) indicates no significant difference (Tukey’s HSD, *p* < 0.05) between the mean expression of the corresponding genes across the tissues.

Figure 5 shows the DESeq2 normalized, autoscaled RNA-Seq expression profile of protease genes belonging to the A1, C1, C13 and C14 families. The samples can be broadly divided into vegetative tissues (seedling) and grain tissues (germinating grain, pre-anthesis ovary, young grain, mid-length grain, full-length grain and mature grain). The hierarchically clustered expression profile revealed clusters of A1, C1 and C13 proteases that are specifically expressed in developing *Brachypodium* grain tissues. Although proteases belonging to the C14 family are expressed in grain tissues, they were also highly expressed in vegetative tissues and none was judged to be grain specific. Clusters of proteases that are highly expressed during the period of nucellus and mesocarp PCD were identified in A1, C1 and C13 families (highlighted yellow in Figure 5a–c). Additionally, clusters of genes that are highly expressed in the endosperm can be distinguished in A1 and C1 (highlighted green in Figure 5a,b). Based on the expression profile, we identified putative grain specific genes from A1, C1 and C13 (Table 1). We also selected one representative gene from each protease family (Figure 5 red rectangles) and validated their expression using quantitative RT-PCR (Figure 6). The results agree with the RNA-Seq data.

## 3. Discussion

Plant PCD research in the past three decades has demonstrated the importance of timely death of certain cells for the normal development of plants. Current knowledge of PCD in developing grains is mainly derived from studies of domesticated species.

In this study, we investigated grain PCD in a wild species, *Brachypodium*. While the general features of PCD are similar between developing cereals and *Brachypodium* grains, we observed subtle differences in timing, pattern and progression of PCD between cereals and *Brachypodium* grains. For example, we detected PCD in the pre-anthesis ovary of *Brachypodium* (Figure 1a). PCD in pre-anthesis ovaries has not been previously reported in other species. A possible reason is that most studies focus on PCD post-fertilization. Our results suggest that PCD is activated in *Brachypodium* nucellar and mesocarp cells before fertilization. Radchuk et al. [5] detected PCD in barley nucellus and mesocarp at anthesis (0 DPA) and 6 DPA, respectively. These results do not exclude the possibility of nucellar PCD already taking place before the sampling time points, but it does suggest that barley mesocarp PCD is initiated between 2 and 6 DPA. It appears that initiation of mesocarp PCD occurs later in barley compared to *Brachypodium*.

*Brachypodium* nucellar disintegration occurs in a centrifugal pattern similar to barley, rice and wheat. However, the rate of nucellar cell disintegration is slow compared to barley. Rapid disintegration of the nucellus is thought to provide growth space and resources for the incipient endosperm in cereals [1]. In maize, early endosperm development around the period of nucellar PCD influences final grain size [41]. It is possible that slow nucellar disintegration in *Brachypodium* impedes its early endosperm development because of reduced space and poor resource supply from degenerated cells. Moreover, *Brachypodium* nucellar epidermis enlarges greatly before undergoing PCD ca. 6 DPA. Interestingly, the nucellar epidermis does not collapse or disintegrate throughout grain development. We have previously shown that the nucellar epidermis serves as an assimilate transport channel towards the endosperm [40]. A similar role is played by rice nucellar epidermis [9] which, in contrast to the *Brachypodium* nucellar epidermis, collapses during grain development.

Conversely, mesocarp disintegration occurs at a faster rate in *Brachypodium* compared to barley. This agrees with the earlier observation that mesocarp PCD is initiated earlier in *Brachypodium* compared to barley. Because barley mesocarp PCD is only detected after endosperm cellularization, Radchuk et al. [5] proposed that barley mesocarp PCD is coordinated with endosperm development. In maize, PCD in the nucellar placento-chalazal region is coordinated with endosperm cellularisation and is completed before the commencement of major grain filling [14]. Okada et al. [42] confirmed coordination between endosperm development and mesocarp PCD in wheat and barley. They showed that mesocarp cells of unfertilized ovaries do not disintegrate. Instead, the mesocarp cells swell laterally and force the lemma and palea apart in a bid to increase the chances of cross pollination and fertilization. Our results suggest that *Brachypodium* lacks this coordination of endosperm development with mesocarp PCD. Furthermore, the rate of degeneration of *Brachypodium* grain mesocarp cells was most rapid in the lateral regions (Figure 2a). This contrasts with the centrifugal mesocarp degeneration pattern reported in barley, rice and wheat [1,5,6,16] Because the endosperm subsequently expands laterally to fill the space left by disintegrated mesocarp cells, we speculate that the pattern of mesocarp disintegration may contribute to the flat shape of *Brachypodium* grains.

*Brachypodium* endosperm PCD was detected at 15 DPA (Figure A1g) and did not progress in any discernible pattern. Random progression of endosperm PCD has also been observed in wheat, whereas maize endosperm PCD proceeds in an organized top-to-base fashion [18,43] The differences in the pattern of endosperm PCD have been attributed to grain size. It has been suggested that while PCD can proceed randomly in the comparatively smaller endosperm of wheat, the larger endosperm of maize requires PCD to be executed in organized manner [18,43] Hence, we conclude that random PCD in *Brachypodium* endosperm may be attributed to its small size.

Proteases are known to contribute to the execution of PCD. Although the details of the contribution of individual genes are yet to be elucidated, the expression profile of several protease genes strongly coincide with PCD in different grain tissues [44]. Such correlative evidence has been used to infer roles for a number of proteases during the PCD of one or more cereal grain tissues. To gain a comprehensive view, we identified all *Brachypodium* genes that belong to protease families whose members have been implicated in PCD of developing grains. The families comprise A1, C1, and C13. There are no available reports linking C14 (metacaspases) to grain PCD. However, we included C14 orthologs in our analyses because members of the family have been characterized in detail in *Arabidopsis* [45]. Furthermore, the expression levels of metacaspase genes were reduced in *Brachypodium* callus treated with 5 and 50 µM 5-Azacitidine [46]. Phylogenetic analyses enabled the identification of *Brachypodium* orthologs within the selected families. RNA-Seq survey of the expression profile of genes in the selected families revealed that the majority of genes in A1 and C1 are lowly expressed in the vegetative and grain tissues sampled. The analyses also revealed novel candidate genes that may be further explored for their roles in grain PCD (Table 1). Nevertheless, mRNA expression of proteases may not always mean activity because proteases are known to be inhibited by cystatins [47].

Remarkably, C13 (VPEs) genes were the most highly expressed in the four families surveyed. Detailed expression analysis of VPEs in barley grain fractions revealed identical expression profiles for *HvVPE2a*, *HvVPE2b* and *HvVPE2d* in nucellar and endosperm fractions [5]. *HvVPE2a* (nucellain) localizes in degenerating nucellar cells [24]. Therefore, these three genes are hypothesized to play major roles in barley grain nucellar PCD. These three genes and their wheat orthologs form a distinct subclade (Figure 4). However, *BdVPE2* is more similar to *HvVPE2c*, which is barely expressed in barley grains. We speculate that the combined activities of barley *HvVPE2a*, *HvVPE2b* and *HvVPE2d* facilitate the rapid disintegration of the barley nucellus. *Brachypodium,* on the other hand, lacks a direct ortholog of these genes and shows a slow nucellar degeneration rate. In addition, *BdVPE2* expression does not overlap with nucellar PCD. This prompts us to suggest that the slow progression of *Brachypodium* nucellar PCD and the persistence of the nucellar epidermis may be partly due to a lack of VPEs that facilitate nucellus disintegration.

## 4. Materials and Methods

### 4.1. Plant Materials

*Brachypodium* Grains (Bd21) Were Obtained from the John Innes Centre, Norwich. Barley grains (cv. Bowman) were Obtained from Division of Plant Sciences, University of Dundee at James Hutton Institute, Invergowrie, Dundee.

*Brachypodium* grains were imbibed on moist filter paper in a Petri dish and left at 5 °C for two days to stratify. They were transferred to room temperature (about 25–27 °C) and left to germinate. After 7 days, the most virile seedlings were transferred to 9:1 Levington M2 Pot and Bedding Compost: Levington Fine Vermiculite mix (Dejex, Donington, UK), in Vacapot 15 on plastic seed trays (H. Smith Plastics, Essex, UK). They were grown in a greenhouse with 16 h daylight and 25 °C temperature. The plants were regularly watered manually. Pre-anthesis ovary samples were collected at the yellow (intact) anther stage. Grain samples were collected from spikes staged at anthesis.

### 4.2. TUNEL Staining

DNA fragmentation was detected using terminal deoxynucleotidyl transferase (TdT) dUTP nick-end labeling (TUNEL). The assay was performed on dewaxed and rehydrated slides treated with proteinase K, according to the manufacture’s protocol of the In Situ Cell Death Detection kit (Roche Diagnostics, Mannheim, Germany). Slides treated with DNase served as the positive control while slides that were not treated with TdT served as the negative control. At least three slides were examined for each grain development stage. Imaging was performed with a Nikon ECLIPSE 80i fluorescence microscope (Nikon, Tokyo, Japan), which has an LED-based excitation source (CoolLED, presicExcite), and using a Nikon Plan Fluor 10 × /0.30 DIC L/N1 objective lens. Fluorescence images were captured with a DS-QiMc cooled CCD camera (Nikon, Tokyo, Japan). Images were previewed, captured and saved using the NIS-Elements Basic Research v3.0 software (Nikon, Tokyo, Japan) in JPEG2000 format.

### 4.3. DNA Isolation and Electrophoresis

Genomic DNA was extracted according to CTAB protocol [48]. DNA was extracted from at least three grains harvested from different plants. DNA extracts were quantified with a NanoDrop spectrophotometer (Thermo Fisher Scientific, Massachusetts, USA). A 15 μL aliquot of 300 ng/μL of DNA was separated on 1% *w*/*v* agarose gel at 100 V for 1.50 h.

### 4.4. Evans Blue Staining

Freehand sections (10–30 μm) of freshly sampled *Brachypodium* grains were immersed in 0.002 dm^3^ Evans Blue solution (0.05% *w*/*v* in water) for 3 min and rinsed in several changes of distilled water with gentle agitation for mortal staining. Stained sections were mounted in 50% *v*/*v* glycerol, then viewed and photographed with a Zeiss Stereo Microscope equipped with a GT-vision GXCAM-5MP digital USB camera and GXCAPTURE software (GT Vision, Suffolk, UK). At least 10 sections were examined for each grain development stage.

### 4.5. Thin Section and Light Microscopy

At 5DPA, 1 mm middle transverse sections were obtained from three grains each of *Brachypodium* and barley (cv. Bowman) (grown in the same conditions as *Brachypodium*) under a dissecting microscope. Sections were fixed in 2.5% *w*/*v* glutaraldehyde in 0.1 M sodium cacodylate buffer pH 7.4 for 3 days at 4 °C with constant gentle agitation and then washed in 0.1 M sodium cacodylate buffer. Further fixation in 1% *v*/*v* aqueous osmium tetroxide was followed by dehydration in a series of increasing ethanol concentrations followed by propylene oxide. The sections were embedded in Spurr’s hard resin and polymerised for 16 h at 60 °C. 400 nm thick sections were obtained with an ultramicrotome, stained for 30 s with 0.01% *w*/*v* toluidine blue, mounted in resin, and imaged with a GX L3200B compound microscope (GT Vision, Suffolk, UK ) equipped with a CMEX-5000 USB2 microscope camera and ImageFocus 4 software (Euromex. Arnhem, Netherlands).

### 4.6. Source and Phylogenetic Analysis of Selected Protease Family Genes

*Brachypodium* protein sequences belonging to protease families A1, C1, C13 and C14 were retrieved using INTERPRO [49] and PANTHER [50] accession numbers specific for each domain (A1: pepsin; IPR001461, PTHR13683), (C1: papain; IPR000668, PTHR12411), (C13: legumain; IPR001096, PTHR12000), (C14: caspase; PTHR31773, PTHR31810) from the peptidase database MEROPS 12.1 [51]. These accession numbers were also used to blast Phytozome v.12 (https://www.phytozome.jgi.doe.gov, accessed on 12 April 2019) and EnsemblePlants (http://plants.ensembl.org, accessed on 13 April 2019). The protein sequences were uploaded into Geneious R10 (Biomatters, Auckland, New Zealand) and filtered by selecting only the unique longest version of the protein sequences. The same approach was used to obtain A1 and C1 protein sequences of *Arabidopsis*, barley, rice and wheat. Protein sequences of C13 and C14 of *Arabidopsis*, barley and rice were gathered from published reports [23,52,53,54,55]. Further information on sequences used in phylogenetic analysis is provided in Table A2. Phylogenetic analysis was performed in MEGA6 [56] and Geneious R10. Alignment was performed using MAFFT v7.308 [57] under the following parameters: BLOSUM62 as the scoring matrix, Gap open penalty of 1.53 and an offset value of 0.123. The phylogenetic tree of C13 was constructed using the maximum likelihood method based on the Le_Gascuel_2008 model [58]. The phylogenetic tree of C14 was constructed using the maximum likelihood method based on the Whelan and Goldman model [59].

### 4.7. RNA-Seq Data Source, Processing and Expression Analysis of Protease Genes

Total RNA was extracted from eight vegetative and grain tissues of *Brachypodium* using the Plant Total RNA Kit (Sigma-Aldrich, Hoddesdon, UK). Each tissue had three biological replicates, giving a total of 24 libraries. Randomly primed cDNA libraries were created and sequenced by GATC Biotech using Illumina HiSeq. About 30 million 50 bp single reads were generated per library. Data quality was checked with FastQC and found satisfactory, so the reads were not trimmed. Reads were mapped with STAR v2.5.2b [60] to *Brachypodium distachyon* v3.0.dna.toplevel.fa, downloaded from Phytozome v.12. Mapped reads were counted with the featureCount function from the Rsubread v 1.22.2 package [61]. Raw read counts were normalized with DEseq2 v1.6.3 [62]. Reads were scaled using the unit variance scaling method. The expression profile of protease gene families in the RNA-Seq data was visualized with the R package ComplexHeatmap v1.99.8 [63]. The raw RNA-Seq data is publicly available at E-MTAB-7607 (http://ebi.ac.uk/arrayexpress, accessed on 31 January 2019).

### 4.8. mRNA In Situ Hybridization

At least three samples for each stage of grain development were harvested and prepared for mRNA in situ hybridization as described previously [28,64]. The probe template consisted of a *BdMADS29* cDNA fragment amplified with gene specific primers (see Table A3 for primer sequences) and transcribed in vitro with T7 RNA polymerase (Bioline.com, Memphis, USA).

### 4.9. Real-Time Quantitative PCR 

Quantitative RT-PCR reactions were carried out using SYBR Green (SensiMix SYBR Low-ROX Kit) (Bioline.com, Memphis, USA) on a 7500 MicroAmp Fast Optical 96-Well Reaction Plate (Applied Biosystems, Massachusetts, USA) with the following reaction mix: 5 µL of 2× SensiMix SYBR Low-ROX, 0.1 µL of 25 µM forward/reverse primers, 3.8 µL of DNase-free H_2_O, and 1 µL of cDNA template. The PCR conditions were: 95 °C for 10 min, 40 cycles of 95 °C for 15 s, 65 °C for 15 s and 72 °C for 15 s. Samples were normalized using *BdACT7* as reference (*Bradi_4g41850v3*). The ΔC_T_ value for each sample was obtained from the difference between the C_T_ values of the reference and target gene. The relative expression levels were subsequently determined using the equation: RQ = 2^−ΔΔCT (sample)^ as described in Livak and Schmittgen [65], using 7500 Software v2.0.6 (Applied Biosystems, Massachusetts, USA). Primer sequences are provided in Table A2. The results are mean values from three experiments performed with cDNA from three biological replicates.

## 5. Conclusions

Our study provides the first detailed description of grain PCD in a non-domesticated small-grained grass, *Brachypodium*. The results and discussion highlight similarities and, crucially, differences in the timing, pattern and progression of grain PCD between *Brachypodium* and cereals. It appears that the rapid degeneration of the nucellar tissues after fertilization in cereals stimulates rapid endosperm expansion, resulting in large grains. We propose that the reduced expansion stimulus offered by the slower degeneration of the *Brachypodium* nucellus contributes to its small grain size. In support of this view, we show that *Brachypodium* has just one (*BdVPE2*) ortholog of nucellus-degenerating VPE, whereas barley has four and wheat has fourteen. Our results also link the patterns of mesocarp PCD with grain shape, and the lateral pattern of mesocarp degeneration appears to contribute to the dorsi-ventrally flattened shape of *Brachypodium* grain.

## Figures and Tables

**Table 1:** Putative *Brachypodium* grain-specific PCD proteases.

Family	Family	Nucellar	Mesocarp	Endosperm
A1	*BRADI1g42930*	-	-	YES
A1	*BRADI2g45800*	-	-	YES
A1	*BRADI1g49410*	YES	YES	-
A1	*BRADI5g26620*	YES	YES	-
A1	*BRADI2g02140*	-	-	YES
C1	*BRADI2g08300*	-	YES	YES
C13	*BRADI5g16960* (*BdVPE2*)	-	YES	YES

## Data Availability

*Brachypodium* grain RNA-Seq data is publicly available at E-MTAB-7607 (http://ebi.ac.uk/arrayexpress, accessed on 31 January 2019).

## Appendix B. Tables

**Table A1:** Numbers of genes found in each species for each family.

			*Brachypodium distachyon*	*Hordeum vulgare*	*Arabidopsis thaliana*	*Oryza sativa*	*Triticum aestivum*
families	Uniprot ID	Panther ID					
A1 pepsin	IPR001461	PTHR13683	93	128	69	102	450
C1 papain	IPR000668	PTHR12411	48	65	39	34	185
C13 nucellain	IPR001096	PTHR12000	5	8	5	6	36
C14 metacaspase	PTHR31773	PTHR31810	10	10	9	10	35

**Table A2:** Primer sequences used in this study.

Gene Name	Gene ID	Polarity	Amplicon Size (bp)	Primer Sequences (5′ to 3′)	Start-End
*BdACT7*	*Bradi_4g41850v3*	Forward	101 bp	GTGACCTAACTGACTGCTTGAT	(705–726)
		Reverse		GAGCTTCTCCTTGATATCCCTTAC	(782–805)
*BdA1 pepsin*	*BRADI_1g42930v3*	Forward	117 bp	GGCACGTCAACAAACTTCCT	(1494–1413)
		Reverse		CGGTTCACCATCATTTACCC	(1591–1610)
*BdC1 papain*	*BRADI_2g08300v3*	Forward	139 bp	CATGATGAGCTCTGATGCCTAT	(1226–1247)
		Reverse		GTGGTGGTGTACTGACATACTG	(1343–1364)
*BdC13 nucellain*	*BRADI_5g16960v3*	Forward	96 bp	GCAGGACAATGCCCTCTGATTT	(2305–2326)
		Reverse		GGACTCTAGGAGGATCACAACCTTAC	(2375–2400)
*BdC14 metacaspase*	*BRADI_2g52470v3*	Forward	96 bp	TTCAGAGTGCTGGTGAGGTTTATG	(1222–1245)
		Reverse		CTGCTGATGTTTGGCTGGTTTG	(1296–1317)
*BdMADS29*	*BRADI_3g05260v3*	Forward	377 bp	AACACTCTCCTGTGCCGCAT	(1083–1102)
		Reverse		CCTCCACCGTGACCTTCTTA	(1440–1459)
		R-T7		GAATTGTAATACGACTCACTATAGGGCCTCCACCGTGACCTTCTTA	

**Table A3:** Gene names and IDs used in Figure 4 and Figure A5, grouped in C13 and C14 families for each specie.

Species	Family	Gene Name	Gene ID	UniProt	Ref
*Arabidopsis thaliana*	C13	*At-gamma-VPE*	*AT4G32940*	A0A178UU68	[66]
		*At-beta-VPE*	*AT1G62710*	A0A178W0Z7	
		*At-alpha-VPE*	*AT2G25940*	A0A178VQP4	
		*At-delta-VPE*	*AT3G20210*	A0A178VB13	
		*At-delta-VPE variant*	*AT1G08750*	A0A178WNN3	
	C14	*AtMCAs1*	*At1g02170*	A0A178W8H4	[67]
		*AtMCAs2*	*At4g25110*	A0A178V2G1	
		*AtMCAs3*	*At5g64240*	F4KDK6	
		*AtMCAs4*	*At1g79340*	A0A178WK95	
		*AtMCAs5*	*At1g79330*	A0A178WIC7	
		*AtMCAs6*	*At1g79320*	A0A178W108	
		*AtMCAs7*	*At1g79310*	A0A178WN22	
		*AtMCAs8*	*At1g16420*	A0A178WDC5	
		*AtMCAs9*	*At5g04200*	A0A178U6S6	
*Oryza sativa*	C13	*OsVPE1/GLUP3*	*Os04g0537900*	Q84LM2	[68]
		*OsVPE2*	*Os01g0559600*	Q7F1B4	
		*OsVPE3*	*Os02g0644000*	Q8GS39	
		*OsVPE4a*	*Os05g0593900*	Q6L4R2	
		*OsVPE4b*	*Os06g0105100*	Q9LWZ3	our study
		*OsVPE5*	*Os02g0219400*	Q6Z6K3	[55]
	C14	*OsMC1*	*Os03g0388900*	Q75LQ1	[66,69]
		*OsMC2*	*Os03g0389400*	A0A0P0VY86	
		*OsMC3*	*Os03g0389000*	A0A0P0VY92	
		*OsMC4*	*Os05g0496400*	A0A0P0WP05	
		*OsMC5*	*Os05g0496500*	Q84VF0	
		*OsMC6*	*Os01g0799900*	Q8LJ88	
		*OsMC7*	*Os11g0134700*	Q2RAW9	
		*OsMC8*	*Os03g0389100*	Q75LQ8	
		*OsMC9*	*Os03g0389501*	A0A0P0VYB1	our study
		*OsMC10*	*Os10g0565100*	A0A0P0XYC7	
*Hordeum vulgare*	C13	*HvLeg-1/HvVPE1*	*HORVU6Hr1G060990*	B4ESD9	[5,23]
		*HvLeg-2/HvVPE2b*	*HORVU2Hr1G092080*	B4ESE0	
		*HvLeg-3/HvVPE2d*	*HORVU2Hr1G091880*	B4ESE1	
		*HvLeg-4/HvVPE3*	*HORVU3Hr1G048520*	B4ESE2	
		*HvLeg-5/HvVPE4*	*HORVU5Hr1G066250*	B4ESE3	
		*HvLeg-6/HvVPE2a, Nucellain*	*HORVU2Hr1G092080*	E5AXU4	
		*HvLeg-7/HvVPE2c*		E5AXU6	
		*HvLeg-8*		F2DJF5	
	C14	*HvMC1*	*HORVU3Hr1G095700*	A0A287MDW0	[52]
		*HvMC2*	*HORVU4Hr1G090860*	A0A287Q5C5	
		*HvMC3*	*HORVU1Hr1G055210*	A0A287FP58	
		*HvMC4*	*HORVU3Hr1G020830*	A0A287KD76	
		*HvMC5*	*HORVU1Hr1G071130*	A0A287G509	
		*HvMC6*	*HORVU5Hr1G023940*	A0A287QP80	
		*HvMC7*	*HORVU3Hr1G078270*	A0A287LT80	
		*HvMC8*	*HORVU5Hr1G044610*	A0A287R060	
		*HvMC9*	*HORVU4Hr1G011900*	A0A287N8J8	
		*HvMC10*	*HORVU1Hr1G067230*	A0A287G1 × 9	
*Brachypodium distachyon*	C13	*BdVPE1*	*BRADI_3g50100v3*	I1IC16	our study
		*BdVPE2*	*BRADI_5g16960v3*	I1J043	
		*BdVPE3*	*BRADI_2g41270v3*	I1HNN1	
		*BdVPE4*	*BRADI_4g30110v3*	I1IQ27	
		*BdVPE5*	*BRADI_3g08190v3*	I1HYS5	
	C14	*BdMC1*	*Bradi_3g33722v3*	A0A2K2D0W7	[66]
		*BdMC2*	*Bradi_1g60762v3*	I1H4W5	
		*BdMC3*	*Bradi_1g60756v3*	I1H4W4	
		*BdMC4*	*Bradi_1g60800v3*	I1H4W9	
		*BdMC5*	*Bradi_1g60787v3*	I1H4W8	
		*BdMC6*	*Bradi_1g60777v3*	I1H4W7	
		*BdMC7*	*Bradi_2g21100v3*	I1HI23	
		*BdMC8*	*Bradi_2g21110v3*	I1HI24	
		*BdMC9*	*Bradi_2g52470v3*	I1HSI1	
		*BdMC10*	*Bradi_2g50480v3*	I1HRT2	

## References

[1] Domínguez F., Cejudo F.J. (2014). Programmed cell death (PCD): An essential process of cereal seed development and germination. Front. Plant Sci..

[2] Morrison I.N., O’Brien T.P., Kuo J. (1978). Initital cellularization and differentiation of the aleurone cells in the ventral region of the developing wheat grain. Planta.

[3] Norstog K. (1974). Nucellus During Early Embryogeny in Barley: Fine Structure. Int. J. Plant Sci..

[4] Chen J., Yi Q., Song Q., Gu Y., Zhang J., Hu Y., Liu H., Liu Y., Yu G., Huang Y. (2015). A highly efficient maize nucellus protoplast system for transient gene expression and studying programmed cell death-related processes. Plant Cell Rep..

[5] Radchuk V., Weier D., Radchuk R., Weschke W., Weber H. (2011). Development of Maternal Seed Tissue in Barley is Medi-ated by Regulated Cell Expansion and Cell Disintegration and Coordinated with Endosperm Growth. J. Exp. Bot..

[6] Yin L., Xue H. (2012). The MADS29 Transcription Factor Regulates the Degradation of the Nucellus and the Nucellar Projection during Rice Seed Development. Plant Cell.

[7] Evers A.D., Reed M. (1988). Some Novel Observations by Scanning Electron Microscopy. Cereal. Chem..

[8] Freeman P.L., Palmer G.H. (1984). THE STRUCTURE OF THE PERICARP AND TESTA OF BARLEY. J. Inst. Brew..

[9] Oparka K.J., Gates P. (1981). Transport of assimilates in the developing caryopsis of rice (*Oryza sativa* L.). Planta.

[10] Wang H.L., Offler C.E., Patrick J.W. (1994). Nucellar projection transfer cells in the developing wheat grain. Protoplasma.

[11] Domínguez F., Cejudo F.J. (1998). Germination-related genes encoding proteolytic enzymes are expressed in the nucellus of developing wheat grains. Plant J..

[12] Ellis J.R., Chaffey N.J. (1987). Structural Differentiation of the Nucellar Epidermis in the Caryopsis of Rice (*Oryza sativa*). Ann. Bot..

[13] Wu X., Liu J., Li D., Liu C.-M. (2016). Rice caryopsis development I: Dynamic changes in different cell layers. J. Integr. Plant Biol..

[14] Kladnik A., Chamusco K., Dermastia M., Chourey P. (2004). Evidence of Programmed Cell Death in Post-Phloem Transport Cells of the Maternal Pedicel Tissue in Developing Caryopsis of Maize. Plant Physiol..

[15] Thiel J., Weier D., Sreenivasulu N., Strickert M., Weichert N., Melzer M., Czauderna T., Wobus U., Weber H., Weschke W. (2008). Different Hormonal Regulation of Cellular Differentiation and Function in Nucellar Projection and Endosperm Transfer Cells: A Microdissection-Based Transcriptome Study of Young Barley Grains. Plant Physiol..

[16] Domínguez F., Moreno J., Cejudo F.J. (2001). The nucellus degenerates by a process of programmed cell death during the early stages of wheat grain development. Planta.

[17] Kobayashi H., Ikeda T.M., Nagata K. (2013). Spatial and temporal progress of programmed cell death in the developing starchy endosperm of rice. Planta.

[18] Young T.E., Gallie D.R., DeMason D.A. (1997). Ethylene-Mediated Programmed Cell Death during Maize Endosperm Development of Wild-Type and Shrunken2 Genotypes. Plant Physiol..

[19] Bi X., Khush G.S., Bennett J. (2005). The Rice Nucellin Gene Ortholog OsAsp1 Encodes an Active Aspartic Protease Without a Plant-specific Insert and is Strongly Expressed in Early Embryo. Plant Cell Physiol..

[20] Chen F., Foolad M.R. (1997). Molecular Organization of a Gene in Barley which Encodes a Protein Similar to Aspartic Protease and its Specific Expression in Nucellar Cells during Degeneration. Plant Mol. Biol..

[21] Asakura T., Watanabe H., Abe K., Arai S. (1995). Rice Aspartic Proteinase, *Oryzasin*, Expressed During Seed Ripening and Germination, has a Gene Organization Distinct from Those of Animal and Microbial Aspartic Proteinases. JBIC J. Biol. Inorg. Chem..

[22] Borén M., Höglund A., Bozhkov P., Jansson C. (2006). Developmental Regulation of a VEIDase Caspase-Like Proteolytic Activity in Barley Caryopsis. J. Exp. Bot..

[23] Julián I., Gandullo J., Santos-Silva L.K., Diaz I., Martinez M. (2013). Phylogenetically distant barley legumains have a role in both seed and vegetative tissues. J. Exp. Bot..

[24] Linnestad C., Doan D.N., Brown R.C., Lemmon B.E., Meyer D.J., Jung R., Olsen O.-A. (1998). Nucellain, a Barley Homolog of the Dicot Vacuolar-Processing Protease, Is Localized in Nucellar Cell Walls. Plant Physiol..

[25] Sreenivasulu N., Radchuk V., Strickert M., Miersch O., Weschke W., Wobus U. (2006). Gene Expression Patterns Reveal Tis-sue-specific Signaling Networks Controlling Programmed Cell Death and ABA-regulated Maturation in Developing Barley Seeds. Plant J..

[26] Tran V., Weier D., Radchuk R., Thiel J., Radchuk V. (2014). Caspase-Like Activities Accompany Programmed Cell Death Events in Developing Barley Grains. PLoS ONE.

[27] Drea S., Leader D.J., Arnold B.C., Shaw P., Dolan L., Doonan J.H. (2005). Systematic Spatial Analysis of Gene Expression during Wheat Caryopsis Development. Plant Cell.

[28] Opanowicz M., Hands P., Betts D., Parker M.L., Toole G.A., Mills E.C., Doonan J.H., Drea S. (2011). Endosperm Development in *Brachypodium Distachyon*. J. Exp. Bot..

[29] Yang X., Wu F., Lin X., Du X., Chong K., Gramzow L., Schilling S., Becker A., Theißen G., Meng Z. (2012). Live and Let Die-the Bsister MADS-Box Gene OsMADS29 Controls the Degeneration of Cells in Maternal Tissues during Seed Development of Rice (*Oryza Sativa*). PLoS ONE.

[30] Radchuk V., Tran V., Radchuk R., Diaz-Mendoza M., Weier D., Fuchs J., Riewe D., Hensel G., Kumlehn J., Munz E. (2017). Vacuolar processing enzyme 4 contributes to maternal control of grain size in barley by executing programmed cell death in the pericarp. New Phytol..

[31] Nayar S., Sharma R., Tyagi A.K., Kapoor S. (2013). Functional delineation of rice MADS29 reveals its role in embryo and endosperm development by affecting hormone homeostasis. J. Exp. Bot..

[32] Draper J., Mur L.A., Jenkins G., Ghosh-Biswas G.C., Bablak P., Hasterok R., Routledge A.P. (2001). *Brachypodium Distachyon*. A New Model System for Functional Genomics in Grasses. Plant Physiol..

[33] Trafford K., Haleux P., Henderson M., Parker M., Shirley N.J., Tucker M.R., Fincher G.B., Burton R.A. (2013). Grain Development in *Brachypodium* and Other Grasses: Possible Interactions between Cell Expansion, Starch Deposition, and Cell-Wall Synthesis. J. Exp. Bot..

[34] Francin-Allami M., Lollier V., Pavlovic M., San Clemente H., Rogniaux H., Jamet E., Guillon F., Larré C. (2016). Under-standing the Remodelling of Cell Walls during *Brachypodium Distachyon* Grain Development through a Sub-Cellular Quantitative Proteomic Approach. Proteomes.

[35] Francin-Allami M., Alvarado C., Daniel S., Geairon A., Saulnier L., Guillon F. (2019). Spatial and temporal distribution of cell wall polysaccharides during grain development of *Brachypodium distachyon*. Plant Sci..

[36] Guillon F., Larre C., Petipas F., Berger A., Moussawi J., Rogniaux H., Santoni A., Saulnier L., Jamme F., Miquel M. (2011). A comprehensive overview of grain development in *Brachypodium distachyon* variety Bd21. J. Exp. Bot..

[37] Hands P., Kourmpetli S., Sharples D., Harris R.G., Drea S. (2012). Analysis of grain characters in temperate grasses reveals distinctive patterns of endosperm organization associated with grain shape. J. Exp. Bot..

[38] Hands P., Drea S. (2012). A comparative view of grain development in *Brachypodium distachyon*. J. Cereal. Sci..

[39] Kourmpetli S., Drea S. (2013). The fruit, the whole fruit, and everything about the fruit. J. Exp. Bot..

[40] Solomon C.U., Drea S. (2019). Delineation of post-phloem assimilate transport pathway into developing caryopsis of *Brachypodium distachyon*. bioRxiv.

[41] Leroux B.M., Goodyke A.J., Schumacher K.I., Abbott C.P., Clore A.M., Yadegari R., Larkins B.A., Dannenhoffer J. (2014). Maize early endosperm growth and development: From fertilization through cell type differentiation. Am. J. Bot..

[42] Okada T., Ridma M., Jayasinghe J.E.A., Nansamba M., Baes M., Warner P., Warner A., Correia D., Nguyen V., Whitford R. (2018). Unfertilized ovary pushes wheat flower open for cross-pollination. J. Exp. Bot..

[43] Young T.E., Gallie D.R. (1999). Analysis of programmed cell death in wheat endosperm reveals differences in endosperm development between cereals. Plant Mol. Biol..

[44] Buono R.A., Hudecek R., Nowack M.K. (2019). Plant proteases during developmental programmed cell death. J. Exp. Bot..

[45] Tsiatsiani L., Van Breusegem F., Gallois P., Zavialov A., Lam E., Bozhkov P.V. (2011). Metacaspases. Cell Death Differ..

[46] Betekhtin A., Milewska-Hendel A., Chajec L., Rojek-Jelonek L., Nowak K., Kwasniewska J., Wolny E., Kurczynska E., Hasterok R. (2018). 5-Azacitidine induces cell death in a tissue culture of *Brachypodium* Dis-tachyon. Int. J. Mol. Sci..

[47] Subburaj S., Zhu D., Li X., Hu Y., Yan Y. (2017). Molecular Characterization and Expression Profiling of *Brachypodium distachyon* L. Cystatin Genes Reveal High Evolutionary Conservation and Functional Divergence in Response to Abiotic Stress. Front. Plant Sci..

[48] Porebski S., Bailey L.G., Baum B.R. (1997). Modification of a CTAB DNA Extraction Protocol for Plants Containing High Polysaccharide and Polyphenol Components. Plant Mol. Biol. Rep..

[49] Mitchell A.L., Attwood T.K., Babbitt P.C., Blum M., Bork P., Bridge A., Brown S.D., Chang H.-Y., El-Gebali S., Fraser M.I. (2019). InterPro in 2019: Improving coverage, classification and access to protein sequence annotations. Nucleic Acids Res..

[50] Thomas P.D., Campbell M.J., Kejariwal A., Mi H., Karlak B., Daverman R., Diemer K., Muruganujan A., Narechania A. (2003). PANTHER: A library of protein families and subfamilies indexed by function. Genome Res..

[51] Rawlings N.D., Waller M., Barrett A.J., Bateman A. (2014). Merops: The database of proteolytic enzymes, their substrates and in-hibitors. Nucleic Acids Res..

[52] Bostancioglu S.M., Tombuloglu G., Tombuloglu H. (2018). Genome-wide identification of barley MCs (metacaspases) and their possible roles in boron-induced programmed cell death. Mol. Biol. Rep..

[53] Rocha A.J., Soares E.L., Costa J.H., Costa W.L., Soares A.A., Nogueira F., Domont G.B., Campos F. (2013). Differential expression of *cysteine* peptidase genes in the inner integument and endosperm of developing seeds of Jatropha curcas L. (Euphorbiaceae). Plant Sci..

[54] Vercammen D., van de Cotte B., de Jaeger G., Eeckhout D., Casteels P., Vandepoele K., Vandenberghe I., Van Beeumen J., Inzeé D., Van Breusegem F. (2004). Type II Metacaspases Atmc4 and Atmc9 of Arabidopsis thaliana cleave substrates after arginine and lysine. J. Biol. Chem..

[55] Wang W., Zhou X.M., Xiong H.X., Mao W.Y., Zhao P., Sun M.X. (2018). Papain-like and legumain-like proteases in rice: Genome-wide identification, comprehensive gene feature characterization and expression analysis. BMC Plant Biol..

[56] Tamura K., Stecher G., Peterson D., Filipski A., Kumar S. (2013). MEGA6: Molecular evolutionary genetics analysis version 6.0. Mol. Biol. Evol..

[57] Katoh K., Standley D.M. (2013). MAFFT multiple sequence alignment software version 7: Improvements in performance and usability. Mol. Biol. Evol..

[58] Le S.Q., Gascuel O. (2008). An improved general amino acid replacement matrix. Mol. Biol. Evol..

[59] Whelan S., Goldman N. (2001). A General Empirical Model of Protein Evolution Derived from Multiple Protein Families Using a Maximum-Likelihood Approach. Mol. Biol. Evol..

[60] Dobin A., Davis C.A., Schlesinger F., Drenkow J., Zaleski C., Jha S., Batut P., Chaisson M., Gingeras T.R. (2013). STAR: Ultrafast universal RNA-seq aligner. Bioinformatics.

[61] Liao Y., Smyth G.K., Shi W. (2019). The R package Rsubread is easier, faster, cheaper and better for alignment and quantification of RNA sequencing reads. Nucleic Acids Res..

[62] Love M.I., Huber W., Anders S. (2014). Moderated estimation of fold change and dispersion for RNA-seq data with DESeq2. Genome Biol..

[63] Gu Z., Eils R., Schlesner M. (2016). Complex heatmaps reveal patterns and correlations in multidimensional genomic data. Bioinformatics.

[64] Drea S., Corsar J., Crawford B., Shaw P., Dolan L., Doonan J.H. (2005). A streamlined method for systematic, high resolution in situ analysis of mRNA distribution in plants. Plant Methods.

[65] Livak K.J., Schmittgen T.D. (2001). Analysis of relative gene expression data using real-time quantitative PCR and the 2(-Delta C(T)) Method. Methods.

[66] Kinoshita T., Nishimura M., Hara-Nishimura I. (1995). Homologues of a vacuolar processing enzyme that are expressed in different organs in Arabidopsis thaliana. Plant Mol. Biol..

[67] Fagundes D., Bohn B., Cabreira C., Leipelt F., Dias N.C.F., Bodanesezanettini M.H., Cagliari A. (2015). Caspases in plants: Metacaspase gene family in plant stress responses. Funct. Integr. Genom..

[68] Deng M., Bian H., Xie Y., Kim Y., Wang W., Lin E., Zhu M. (2011). Bcl-2 suppresses hydrogen peroxide-induced pro-grammed cell death via OsVPE2 and OsVPE3, but not via OsVPE1 and OsVPE4, in rice. FEBS J..

[69] Zhang D., Yuan Z. (2014). Molecular Control of Grass Inflorescence Development. Annu. Rev. Plant Biol..

